# Supplementary material for: Active, Reactive, and Apparent Power in Dielectrophoresis: Force Corrections from the Capacitive Charging Work on Suspensions Described by Maxwell-Wagner’s Mixing Equation
Source: Micromachines (Basel). 2021 Jun 23;12(7):738. doi: 10.3390/mi12070738 (PMC8305549; doi:10.3390/mi12070738)

Derivations on

"Active, reactive and apparent power in dielectrophoresis: Force corrections from the capacitive charging work on suspensions described by Maxwell-Wagner's mixing equation"

Jan Gimsa, Department of Biophysics, Rostock University, September 2020 ... May 2021

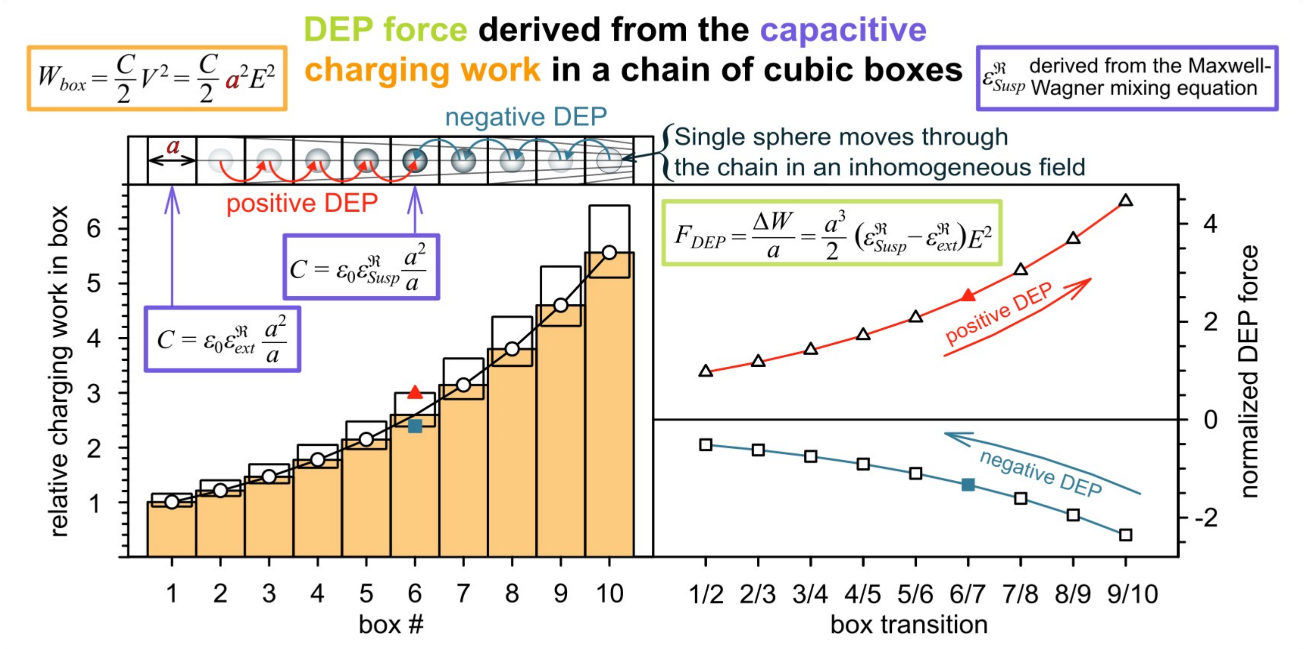

Abstract

The DEP force results from the work of charging two box capacitors (N and N1) in an inhomogeneous field with constant gradient. The capacitance of the boxes is changed by the presence of a single object on which the DEP force acts. To model the DEP force, the box capacitors are discharged before the field-free transfer of the object between the box centers. After recharging, the DEP force is calculated from the total electrical work. The Maxwell-Wagner mixing equation is used to calculate the box capacitance in the presence of the object. Here, homogeneous spherical objects (0-shell sphere: 0SS) are considered. However, the approach can be extended to homogeneous ellipsoids (0SE), cylinders (0SC), single-shell (1SS, 1SE, etc.) and multi-shell objects. The work performed to charge boxes N and N1 is approximated in each box by constant fields corresponding to the field strengths at the centers of the two boxes. Both box capacitors, N and N1, have electrode areas a•b at distance 'a' (in 2D, b=1m). The box volume is Vbox=a^2·b with b=a (cubic box) or b=1m (cuboid box). The object volume is V0= 4/3·π·r^3=p•Vbox, where p is the volume fraction. The box width 'a' is also the reference length for calculating the field gradient from the constant fields assumed in the two neighboring boxes. The work, dissipation and force parameters are derived in terms of their physical units and, for comparison, as the Clausius-Mossotti factor (CMF).

Disclaimer: The manuscript "Active, reactive and apparent power in dielectrophoresis: Force corrections from the capacitive charging work on suspensions described by Maxwell-Wagner's mixing equation" is in main parts based on this program, which is intended to explain the general ideas. In order to reproduce the results of the associated manuscript, parameters such as the field gradient, etc. must be adapted to the special problem under consideration. Some of the results need to be further processed (see explanation in the manuscript).

This program code was developed with Maple 2018.1, Waterloo Maple Inc. It contributes to:

1. A capacitor charging-cycle model for the derivation of DEP force
2. A new Clausius-Mossotti factor derived from Maxwell-Wagner's mixing equation
3. Defining the complex, active and reactive contributions to DEP
4. DEP as a conditioned polarization process
5. Show a possible relation of DEP to the law of maximum entropy production

Permittivities and conductivities of media

restart;

$\epsilon e := \epsilon e\_re - \frac{I\cdot\sigma e\_re}{\epsilon 0\cdot\omega} : \epsilon i := \epsilon i\_re - \frac{I\cdot\sigma i\_re}{\epsilon 0\cdot\omega} :$ # *complex rel. permittivity*

$\sigma e := \sigma e\_re + I\cdot\omega\cdot\epsilon 0\cdot\epsilon e\_re : \sigma i := \sigma i\_re + I\cdot\omega\cdot\epsilon 0\cdot\epsilon i\_re :$ # *complex spec. conductivity*

Clausius-Mossotti factor

General ellipsoid

Permittivity version : $CMF := \frac{\epsilon i - \epsilon e}{\epsilon e + n\cdot(\epsilon i - \epsilon e)}$;

Condutivity version : $CMF := \frac{\sigma i - \sigma e}{\sigma e + n\cdot(\sigma i - \sigma e)}$; # *note the equivalence of the two versions*

For depolarizing coefficient n, see :
Gimsa 2001, Bioelectrochem. 54 : 23 − 31; Maswiwat et al. 2007, J. Phys. D : Appl. Phys. 40 : 914 − 923

Cylinder (or_2D − sphere) : $CMF := 2\cdot\frac{\epsilon i - \epsilon e}{\epsilon i + \epsilon e}$;

1SS with cytoplasmic membrane (finite elements): $CMF := 3 - \frac{9}{2}\cdot\frac{Zi + Zm}{Zi + Zm + Ze}$;

Zi, Zm, Ze are the impedances of internal, membrane and external media elements
For element geometries see: Gimsa & Wachner 1999, Biophysical J. 77: 1316–1326

$CMF := 3\cdot\frac{\sigma i - \sigma e}{\sigma i + 2\cdot\sigma e}$; # *or* $CMF := 3\cdot\frac{\epsilon i - \epsilon e}{\epsilon i + 2\cdot\epsilon e}$; # *for_sphere* $n := \frac{1}{3}$

$$CMF := \frac{3\,(\sigma i\_re + \mathrm{I}\,\omega\,\epsilon 0\,\epsilon i\_re - \sigma e\_re - \mathrm{I}\,\omega\,\epsilon 0\,\epsilon e\_re)}{\sigma i\_re + \mathrm{I}\,\omega\,\epsilon 0\,\epsilon i\_re + 2\,\sigma e\_re + 2\,\mathrm{I}\,\omega\,\epsilon 0\,\epsilon e\_re}$$

Capacities

$C0\_re := \epsilon 0\cdot\epsilon e\_re\cdot\frac{a\cdot b}{a}$: # *without object*

$Cobj\_re := \epsilon 0\cdot\Re(\epsilon S\_act)\cdot\frac{a\cdot b}{a}$: # *with object*

Field gradient

$EN1 := \alpha \cdot EN : \alpha := (1 + ga \cdot a) :$ # approximation of linear field gradient

$EN := \frac{E0}{\sqrt{2}} :$ # introduction of E0 for_comparison with dipole approximation

Charging cycle; physical unit [Ws]

$Wcharge\_N0 := \frac{EN^2 \cdot Vch}{2 \cdot b} \cdot C0\_re : Wcharge\_N10 := \frac{EN1^2 \cdot Vch}{2 \cdot b} \cdot C0\_re :$ # without object

$Wcharge\_Nobj := \frac{EN^2 \cdot Vch}{2 \cdot b} \cdot Cobj\_re : Wcharge\_N1obj := \frac{EN1^2 \cdot Vch}{2 \cdot b} \cdot Cobj\_re :$ # with object

$Wdischarge := -(Wcharge\_Nobj + Wcharge\_N10) :$ # first step of cycle : discharging

second step : field-free object translation

$Wcharge := Wcharge\_N0 + Wcharge\_N1obj :$ # third step : recharging

$Wc := simplify(Wdischarge + Wcharge, symbolic);$ # charging work difference

$$Wc := -\frac{E0^2\ Vch\ ga\ a\ (C0\_re - Cobj\_re)\ (ga\ a + 2)}{4\ b}$$

Energy dispersion difference in cycle; physical unit [W]

$Pdisperse\_N0 := \sigma e\_re \cdot EN^2 \cdot Vch : Pdisperse\_N10 := \sigma e\_re \cdot EN1^2 \cdot Vch :$ # without object

$Pdisperse\_Nobj := \Re(\sigma S\_act) \cdot EN^2 \cdot Vch : Pdisperse\_N1obj := \Re(\sigma S\_act) \cdot EN1^2 \cdot Vch :$ # with object

$Pdisperse0 := Pdisperse\_Nobj + Pdisperse\_N10 :$ # state before transition

$Pdisperse1 := Pdisperse\_N0 + Pdisperse\_N1obj :$ # state after transition

$Pd := simplify(Pdisperse1 - Pdisperse0, symbolic);$ # dispersion difference

$$Pd := -\frac{E0^2\ Vch\ a\ ga\ (\sigma e\_re - \Re(\sigma S\_act))\ (ga\ a + 2)}{2}$$

Simplifications for # $ga \cdot a < 2$:

$Wc := \frac{Vch \cdot ga \cdot a \cdot \epsilon 0 \cdot E0^2 \cdot (\Re(\epsilon S\_act) - \epsilon e\_re)}{2};$

$Pd := Vch \cdot a \cdot E0^2 \cdot (\Re(\sigma S\_act) - \sigma e\_re) :$

multiplication with 1 second yields electrical work (dispersed energy) in_[Ws]

$Wd := Pd \cdot 1;$

$$Wc := \frac{Vch\ ga\ a\ \epsilon 0\ E0^2\ (-\epsilon e\_re + \Re(\epsilon S\_act))}{2}$$

$$Wd := Vch\ a\ E0^2\ (-\sigma e\_re + \Re(\sigma S\_act))$$

Mixing equations

$$\epsilon S\_com := \frac{3+2\cdot p\cdot CMF}{3-p\cdot CMF}\cdot \epsilon e;$$ *# cf. manuscript for complex, active & reactive parameters*

$$\epsilon S\_act := \frac{3+2\cdot p\cdot CMF}{3-p\cdot CMF}\cdot \epsilon e\_re;$$ *# cf. manuscript for ϵe vs. ϵe_re*

$$\epsilon S\_react := \frac{3+2\cdot p\cdot CMF}{3-p\cdot CMF}\cdot\left(-\frac{I\cdot\sigma e\_re}{\epsilon 0\cdot\omega}\right);$$

$$\sigma S\_com := \frac{3+2\cdot p\cdot CMF}{3-p\cdot CMF}\cdot \sigma e;$$ *# cf. manuscript for σe vs. σe_re*

$$\sigma S\_act := \frac{3+2\cdot p\cdot CMF}{3-p\cdot CMF}\cdot \sigma e\_re;$$

$$\sigma S\_react := \frac{3+2\cdot p\cdot CMF}{3-p\cdot CMF}\cdot(I\cdot\omega\cdot\epsilon 0\cdot\epsilon e\_re);$$

$$\epsilon S\_com := \frac{\left(\frac{6\,(\sigma i\_re+\mathrm{I}\,\omega\,\epsilon 0\,\epsilon i\_re-\sigma e\_re-\mathrm{I}\,\omega\,\epsilon 0\,\epsilon e\_re)\,p}{\sigma i\_re+\mathrm{I}\,\omega\,\epsilon 0\,\epsilon i\_re+2\,\sigma e\_re+2\,\mathrm{I}\,\omega\,\epsilon 0\,\epsilon e\_re}+3\right)\left(\epsilon e\_re-\frac{\mathrm{I}\,\sigma e\_re}{\epsilon 0\,\omega}\right)}{-\frac{3\,(\sigma i\_re+\mathrm{I}\,\omega\,\epsilon 0\,\epsilon i\_re-\sigma e\_re-\mathrm{I}\,\omega\,\epsilon 0\,\epsilon e\_re)\,p}{\sigma i\_re+\mathrm{I}\,\omega\,\epsilon 0\,\epsilon i\_re+2\,\sigma e\_re+2\,\mathrm{I}\,\omega\,\epsilon 0\,\epsilon e\_re}+3}$$

$$\epsilon S\_act := \frac{\left(\frac{6\,(\sigma i\_re+\mathrm{I}\,\omega\,\epsilon 0\,\epsilon i\_re-\sigma e\_re-\mathrm{I}\,\omega\,\epsilon 0\,\epsilon e\_re)\,p}{\sigma i\_re+\mathrm{I}\,\omega\,\epsilon 0\,\epsilon i\_re+2\,\sigma e\_re+2\,\mathrm{I}\,\omega\,\epsilon 0\,\epsilon e\_re}+3\right)\epsilon e\_re}{-\frac{3\,(\sigma i\_re+\mathrm{I}\,\omega\,\epsilon 0\,\epsilon i\_re-\sigma e\_re-\mathrm{I}\,\omega\,\epsilon 0\,\epsilon e\_re)\,p}{\sigma i\_re+\mathrm{I}\,\omega\,\epsilon 0\,\epsilon i\_re+2\,\sigma e\_re+2\,\mathrm{I}\,\omega\,\epsilon 0\,\epsilon e\_re}+3}$$

$$\epsilon S\_react := \frac{-\mathrm{I}\left(\frac{6\,(\sigma i\_re+\mathrm{I}\,\omega\,\epsilon 0\,\epsilon i\_re-\sigma e\_re-\mathrm{I}\,\omega\,\epsilon 0\,\epsilon e\_re)\,p}{\sigma i\_re+\mathrm{I}\,\omega\,\epsilon 0\,\epsilon i\_re+2\,\sigma e\_re+2\,\mathrm{I}\,\omega\,\epsilon 0\,\epsilon e\_re}+3\right)\sigma e\_re}{\left(-\frac{3\,(\sigma i\_re+\mathrm{I}\,\omega\,\epsilon 0\,\epsilon i\_re-\sigma e\_re-\mathrm{I}\,\omega\,\epsilon 0\,\epsilon e\_re)\,p}{\sigma i\_re+\mathrm{I}\,\omega\,\epsilon 0\,\epsilon i\_re+2\,\sigma e\_re+2\,\mathrm{I}\,\omega\,\epsilon 0\,\epsilon e\_re}+3\right)\epsilon 0\,\omega}$$

$$\sigma S\_com := \frac{\left(\frac{6\,(\sigma i\_re+\mathrm{I}\,\omega\,\epsilon 0\,\epsilon i\_re-\sigma e\_re-\mathrm{I}\,\omega\,\epsilon 0\,\epsilon e\_re)\,p}{\sigma i\_re+\mathrm{I}\,\omega\,\epsilon 0\,\epsilon i\_re+2\,\sigma e\_re+2\,\mathrm{I}\,\omega\,\epsilon 0\,\epsilon e\_re}+3\right)(\sigma e\_re+\mathrm{I}\,\omega\,\epsilon 0\,\epsilon e\_re)}{-\frac{3\,(\sigma i\_re+\mathrm{I}\,\omega\,\epsilon 0\,\epsilon i\_re-\sigma e\_re-\mathrm{I}\,\omega\,\epsilon 0\,\epsilon e\_re)\,p}{\sigma i\_re+\mathrm{I}\,\omega\,\epsilon 0\,\epsilon i\_re+2\,\sigma e\_re+2\,\mathrm{I}\,\omega\,\epsilon 0\,\epsilon e\_re}+3}$$

$$\sigma S\_act := \frac{\left(\frac{6\,(\sigma i\_re+\mathrm{I}\,\omega\,\epsilon 0\,\epsilon i\_re-\sigma e\_re-\mathrm{I}\,\omega\,\epsilon 0\,\epsilon e\_re)\,p}{\sigma i\_re+\mathrm{I}\,\omega\,\epsilon 0\,\epsilon i\_re+2\,\sigma e\_re+2\,\mathrm{I}\,\omega\,\epsilon 0\,\epsilon e\_re}+3\right)\sigma e\_re}{-\frac{3\,(\sigma i\_re+\mathrm{I}\,\omega\,\epsilon 0\,\epsilon i\_re-\sigma e\_re-\mathrm{I}\,\omega\,\epsilon 0\,\epsilon e\_re)\,p}{\sigma i\_re+\mathrm{I}\,\omega\,\epsilon 0\,\epsilon i\_re+2\,\sigma e\_re+2\,\mathrm{I}\,\omega\,\epsilon 0\,\epsilon e\_re}+3}$$

$$\sigma S\_react := \frac{\mathrm{I}\left(\frac{6\,(\sigma i\_re+\mathrm{I}\,\omega\,\epsilon 0\,\epsilon i\_re-\sigma e\_re-\mathrm{I}\,\omega\,\epsilon 0\,\epsilon e\_re)\,p}{\sigma i\_re+\mathrm{I}\,\omega\,\epsilon 0\,\epsilon i\_re+2\,\sigma e\_re+2\,\mathrm{I}\,\omega\,\epsilon 0\,\epsilon e\_re}+3\right)\omega\,\epsilon 0\,\epsilon e\_re}{-\frac{3\,(\sigma i\_re+\mathrm{I}\,\omega\,\epsilon 0\,\epsilon i\_re-\sigma e\_re-\mathrm{I}\,\omega\,\epsilon 0\,\epsilon e\_re)\,p}{\sigma i\_re+\mathrm{I}\,\omega\,\epsilon 0\,\epsilon i\_re+2\,\sigma e\_re+2\,\mathrm{I}\,\omega\,\epsilon 0\,\epsilon e\_re}+3}$$

Volume fraction p

$$Vch := \frac{V0}{p} :$$

Forces and force-equivalent dispersion

$$Fc := \frac{Wc}{a} : Fc := collect(simplify(evalc(Fc), symbolic), \omega) ; \text{\# new solution for\_DEP force}$$

$$Fc\_new\_CMF := \frac{Fc}{\epsilon0 \cdot \epsilon e\_re \cdot V0 \cdot \frac{ga}{2} \cdot E0^2} ; \qquad \text{\# new CMF}$$

$$Fc\_p0 := limit(Fc, p = 0) : \qquad \text{\# equivalent to classical DEP force}$$

$$Fc\_CMF := \frac{Fc\_p0}{\epsilon0 \cdot \epsilon e\_re \cdot V0 \cdot \frac{ga}{2} \cdot E0^2} : \text{\# cancelling highlighted prefactor shows equivalence with } Re(CMF)$$

$$Fc\_CMF := evalc(Fc\_CMF) ; \text{\# identical to } \Re\text{(classical CMF)}$$

$$Fd := \frac{Wd}{a} : \text{\# 'force equivalent' of dipersion}$$

$$Fd\_p0 := limit(Fd, p = 0) :$$

$$Fd\_CMF := \frac{Fd\_p0}{\sigma e\_re \cdot V0 \cdot E0^2} : \text{\# cancelling highlighted prefactor shows equivalence with } \Re(\text{classical CMF})$$

$$Fd\_CMF := evalc(Fd\_CMF) : \text{\# identical to } \Re\text{(classical CMF)}$$

$$Fc := -\left(3\, \epsilon0\, \epsilon e\_re\, ga\, \left(\left((\epsilon i\_re - \epsilon e\_re)^2\, \epsilon02\, p - (2\, \epsilon e\_re + \epsilon i\_re)\, (\epsilon i\_re - \epsilon e\_re)\, \epsilon02\right) \omega^2 + (-\sigma i\_re + \sigma e\_re)^2 p + 2\, \sigma e\_re^2 - \sigma i\_re\, \sigma e\_re - \sigma i\_re^2\right) E0^2\, V0\right) \Big/ \left(\left(2\, (\epsilon i\_re - \epsilon e\_re)^2\, \epsilon02\, p^2 - 4\, (2\, \epsilon e\_re + \epsilon i\_re)\, (\epsilon i\_re - \epsilon e\_re)\, \epsilon02\, p + 2\, (2\, \epsilon e\_re + \epsilon i\_re)^2\, \epsilon02\right) \omega^2 + 2\, (-\sigma i\_re + \sigma e\_re)^2\, p^2 + \left(8\, \sigma e\_re^2 - 4\, \sigma i\_re\, \sigma e\_re - 4\, \sigma i\_re^2\right) p + 8 \left(\sigma e\_re + \frac{\sigma i\_re}{2}\right)^2\right)$$

$$Fc\_new\_CMF := -\left(6\, \left(\left((\epsilon i\_re - \epsilon e\_re)^2\, \epsilon02\, p - (2\, \epsilon e\_re + \epsilon i\_re)\, (\epsilon i\_re - \epsilon e\_re)\, \epsilon02\right) \omega^2 + (-\sigma i\_re + \sigma e\_re)^2 p + 2\, \sigma e\_re^2 - \sigma i\_re\, \sigma e\_re - \sigma i\_re^2\right)\right) \Big/ \left(\left(2\, (\epsilon i\_re - \epsilon e\_re)^2\, \epsilon02\, p^2 - 4\, (2\, \epsilon e\_re + \epsilon i\_re)\, (\epsilon i\_re - \epsilon e\_re)\, \epsilon02\, p + 2\, (2\, \epsilon e\_re + \epsilon i\_re)^2\, \epsilon02\right) \omega^2 + 2\, (-\sigma i\_re + \sigma e\_re)^2\, p^2 + \left(8\, \sigma e\_re^2 - 4\, \sigma i\_re\, \sigma e\_re - 4\, \sigma i\_re^2\right) p + 8 \left(\sigma e\_re + \frac{\sigma i\_re}{2}\right)^2\right)$$

$$Fc\_CMF := \frac{-6\, \omega^2\, \epsilon e\_re2\, \epsilon02 + 3\, \omega^2\, \epsilon e\_re\, \epsilon i\_re\, \epsilon02 + 3\, \omega^2\, \epsilon i\_re2\, \epsilon02 - 6\, \sigma e\_re^2 + 3\, \sigma i\_re\, \sigma e\_re + 3\, \sigma i\_re^2}{4\, \omega^2\, \epsilon e\_re2\, \epsilon02 + 4\, \omega^2\, \epsilon e\_re\, \epsilon i\_re\, \epsilon02 + \omega^2\, \epsilon i\_re2\, \epsilon02 + 4\, \sigma e\_re^2 + 4\, \sigma i\_re\, \sigma e\_re + \sigma i\_re^2}$$

Imaginary parts of ROT torque and dispersion

(this is not a derivation; see manuscript)

$Tc := \mathrm{Im}(\epsilon S\_act);\ Tc := simplify(evalc(Tc), symbolic)$:# new torque expression from_capacitive charging

$Tc\_new\_CMF := \frac{Tc}{p \cdot \epsilon e\_re}$;   # imaginary part corresponding to Fc_new_CMF

$Tc\_Nm := \frac{\epsilon 0 \cdot Vch \cdot ga \cdot E0^2}{2} \cdot Tc$: # torque in_[Nm] for_comparison with force in_[N]

$Tc\_CMF := limit\left(\frac{Tc}{p \cdot \epsilon e\_re}, p = 0\right)$: # $\Im$(CMF) from_charging derivation is identical_to $\Im$(classical CMF)

$Tc\_Nm\_ref := \epsilon 0 \cdot \epsilon e\_re \cdot V0 \cdot \frac{ga}{2} \cdot Tc\_CMF;$

$Td := \mathrm{Im}(\sigma S\_act);\ Td := simplify(evalc(Td), symbolic)$:

$Td\_Ws := Vch \cdot E0^2 \cdot \mathrm{Im}(\sigma S)$: # dispersion in suspension box in [Ws]

$Td\_CMF := limit\left(\frac{Td}{p \cdot \sigma e\_re}, p = 0\right)$: # $\Im$(CMF) from_capacitor charging and_dispersion are identical

$$Tc := \Im\left(\frac{\left(\frac{6\,(\sigma i\_re + I\,\omega\,\epsilon 0\,\epsilon i\_re - \sigma e\_re - I\,\omega\,\epsilon 0\,\epsilon e\_re)\,p}{\sigma i\_re + I\,\omega\,\epsilon 0\,\epsilon i\_re + 2\,\sigma e\_re + 2\,I\,\omega\,\epsilon 0\,\epsilon e\_re} + 3\right)\epsilon e\_re}{-\frac{3\,(\sigma i\_re + I\,\omega\,\epsilon 0\,\epsilon i\_re - \sigma e\_re - I\,\omega\,\epsilon 0\,\epsilon e\_re)\,p}{\sigma i\_re + I\,\omega\,\epsilon 0\,\epsilon i\_re + 2\,\sigma e\_re + 2\,I\,\omega\,\epsilon 0\,\epsilon e\_re} + 3}\right)$$

$$Tc\_new\_CMF := (9\,(-\epsilon e\_re\,\sigma i\_re + \epsilon i\_re\,\sigma e\_re)\,\epsilon 0\,\omega) \Big/ \Big((\omega^2\,(\epsilon e\_re - \epsilon i\_re)^2\,\epsilon 02 + (-\sigma i\_re + \sigma e\_re)^2)\,p^2 + \left(4\,(\epsilon e\_re - \epsilon i\_re)\,\omega^2\left(\epsilon e\_re + \frac{\epsilon i\_re}{2}\right)\epsilon 02 + 4\,\sigma e\_re^2 - 2\,\sigma i\_re\,\sigma e\_re - 2\,\sigma i\_re^2\right)p + 4\,\omega^2\left(\epsilon e\_re + \frac{\epsilon i\_re}{2}\right)^2\epsilon 02 + 4\left(\sigma e\_re + \frac{\sigma i\_re}{2}\right)^2\Big)$$

$$Tc\_Nm\_ref := -\frac{9\,\epsilon 02\,\epsilon e\_re\,V0\,ga\,\omega\,(\epsilon e\_re\,\sigma i\_re - \epsilon i\_re\,\sigma e\_re)}{2\,(4\,\omega^2\,\epsilon e\_re2\,\epsilon 02 + 4\,\omega^2\,\epsilon e\_re\,\epsilon i\_re\,\epsilon 02 + \omega^2\,\epsilon i\_re2\,\epsilon 02 + 4\,\sigma e\_re^2 + 4\,\sigma i\_re\,\sigma e\_re + \sigma i\_re^2)}$$

$$Td := \Im\left(\frac{\left(\frac{6\,(\sigma i\_re + I\,\omega\,\epsilon 0\,\epsilon i\_re - \sigma e\_re - I\,\omega\,\epsilon 0\,\epsilon e\_re)\,p}{\sigma i\_re + I\,\omega\,\epsilon 0\,\epsilon i\_re + 2\,\sigma e\_re + 2\,I\,\omega\,\epsilon 0\,\epsilon e\_re} + 3\right)\sigma e\_re}{-\frac{3\,(\sigma i\_re + I\,\omega\,\epsilon 0\,\epsilon i\_re - \sigma e\_re - I\,\omega\,\epsilon 0\,\epsilon e\_re)\,p}{\sigma i\_re + I\,\omega\,\epsilon 0\,\epsilon i\_re + 2\,\sigma e\_re + 2\,I\,\omega\,\epsilon 0\,\epsilon e\_re} + 3}\right)$$

Classical CMF as reference

$F\_class := evalc(\Re(CMF));$ # $F\_class := \epsilon 0\ \epsilon e\_re \cdot V0 \cdot \Re(CMF)\ \frac{ga}{2}\ E0^2;$ # $\Re(CMF)$

$F\_\omega 0\_class := limit(F\_class, \omega = 0) : F\_\omega 0\_class := simplify(evalc(F\_\omega 0\_class));$ # ω0-DEP plateau

$F\_\omega inf\_class := limit(F\_class, \omega = \text{infinity}) : F\_\omega inf\_class := simplify(evalc(F\_\omega inf\_class));$ # ωinf-DEP plateau

$$F\_class := \frac{3\ (\sigma i\_re - \sigma e\_re)\ (\sigma i\_re + 2\ \sigma e\_re)}{(\sigma i\_re + 2\ \sigma e\_re)^2 + (2\ \omega\ \epsilon 0\ \epsilon e\_re + \omega\ \epsilon 0\ \epsilon i\_re)^2} + \frac{3\ (-\omega\ \epsilon 0\ \epsilon e\_re + \omega\ \epsilon 0\ \epsilon i\_re)\ (2\ \omega\ \epsilon 0\ \epsilon e\_re + \omega\ \epsilon 0\ \epsilon i\_re)}{(\sigma i\_re + 2\ \sigma e\_re)^2 + (2\ \omega\ \epsilon 0\ \epsilon e\_re + \omega\ \epsilon 0\ \epsilon i\_re)^2}$$

$$F\_\omega 0\_class := \frac{-3\ \sigma e\_re + 3\ \sigma i\_re}{\sigma i\_re + 2\ \sigma e\_re}$$

$$F\_\omega inf\_class := \frac{-3\ \epsilon e\_re + 3\ \epsilon i\_re}{2\ \epsilon e\_re + \epsilon i\_re}$$

Parameters of 0SS model; general

$\text{omega} := 2 \cdot \text{Pi} \cdot 10^{logf}:$

$\epsilon 0 := 8.854185\text{e}-12 :$

$E := 1 : E0 := 1 :$

$ga := 0.1 \cdot \frac{1}{a}$: # 10 % field increase per box; # ga := 1; # normalized field gradient;

Parameters of 0SS model; box and object volumes

$a := 4e-5 : b := a : Vch := a^2 \cdot b;$ # note : Vch is cancelled out in_final force and_torque equations;

$r := 1e-5 : V0 := \frac{4}{3} \text{Pi} \cdot r^3;$ # object volume

$p := \frac{V0}{Vch};$ # volume fraction (Maxwell-limit: 0.1)

$$Vch := 6.4\ 10^{-14}$$

$$V0 := 4.188790205\ 10^{-15}$$

$$p := 0.06544984695$$

Parameters of 0SS model; media properties

external medium

$\sigma e\_re := 0.1 : \epsilon e\_re := 80 :$

homogeneous spheres;

σi_re:=0.01: εi_re:=800: # set #1 passive

$\sigma i\_re := 1 : \epsilon i\_re := 8 :$ # set #2 active

Plots

Reference: classical CMF (note sign definitions for DEP and ROT)

```
with(plots) :

F_class_plot := plot(Re(CMF), logf = 5 ..10, color = red) :
T_class_plot := plot(-Im(CMF), logf = 5 ..10, color = red) :
F_ω0_class_plot := plot(F_ω0_class, logf = 5 ..10, color = red) :   # plateau level
F_ωinf_class_plot := plot(F_ωinf_class, logf = 5 ..10, color = red) : # plateau level
ComClass := complexplot(Re(CMF) - I·Im(CMF), logf = 2 ..11, color = red) :

display({F_class_plot, F_ω0_class_plot, F_ωinf_class_plot, T_class_plot}, title = "reference CMF") :
display(ComClass, scaling = constrained, labels = [Real_CMF, Imag_CMF], title = "reference CMF") :
```

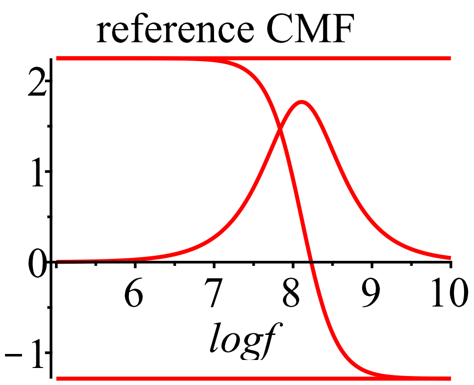

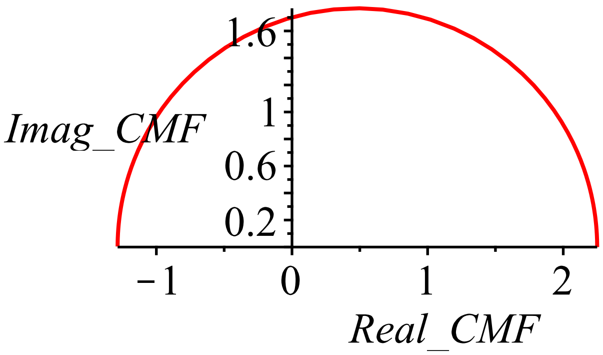

Comparison of final CMF results

red: classical CMF, green: CMF from C-work, blue: CMF from dissipation

```
# CMF derived_from charging work for_p ⇒ 0
Fc_CMF_plot := plot( Fc_CMF, logf = 7.5 ..8.7, color = green ) :   # Re(CMF)
Tc_CMF_plot := plot( -Tc_CMF, logf = 7.5 ..8.7, color = green ) : # Im(CMF)
ComC := complexplot( Fc_CMF - I·Tc_CMF, logf = 2 ..7.8, color = green ) :

# CMF derived_from dissipation for_p ⇒ 0
Fd_CMF_plot := plot( Fd_CMF, logf = 7.5 ..8.7, color = blue ) : # Re(CMF)
Td_CMF_plot := plot( -Td_CMF, logf = 7.5 ..8.7, color = blue ) :# Im( CMF )
ComD := complexplot( Fd_CMF - I·Td_CMF, logf = 8.5 ..11, color = blue ) :

# Display
display( F_ω0_class_plot, F_ωinf_class_plot, F_class_plot, T_class_plot, Fc_CMF_plot, Tc_CMF_plot, title
    = "charging vs. reference CMF" ) :
display( F_ω0_class_plot, F_ωinf_class_plot, F_class_plot, T_class_plot, Fd_CMF_plot, Td_CMF_plot, title
    = "dissipation vs. reference CMF" ) :
display( ComClass, ComC, ComD, scaling = constrained, labels = [ Real_CMF, Imag_CMF ] ) :
```

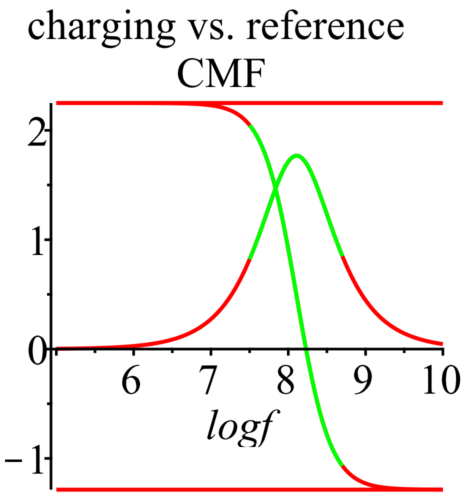

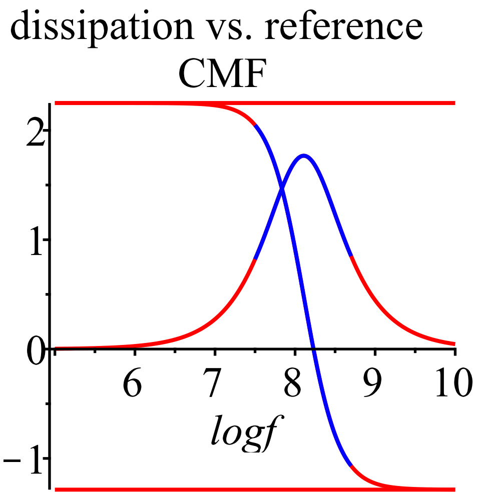

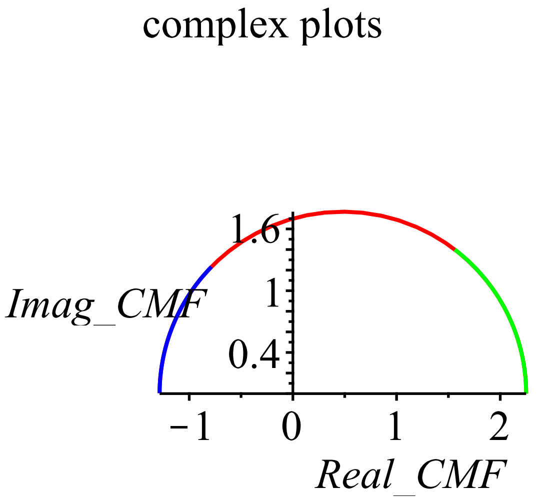

New DEP-force and ROT-torque expressions derived from C-work (green) compared with results after the boundary transition of p⇒0,
i.e. the classical CMF (red)

```
Fc_new_plot := plot(Fc_new_CMF, logf = 5 ..10, color = green) :   # new Re(CMF) from C-work
Tc_new_plot := plot( -Tc_new_CMF, logf = 5 ..10, color = green) : # new Im(CMF) from C-work

Fc_plot := plot(Fc, logf = 5 ..10, color = green) :                    # force corresponding to new CMF
Fc_p0_plot := plot(Fc_p0, logf = 5 ..10, color = red) :               # force corresponding to CMF
Tc_Nm_plot := plot( -Tc_Nm, logf = 5 ..10, color = green) :           # torque corresponding to new CMF
Tc_Nm_ref_plot := plot( -Tc_Nm_ref, logf = 5 ..10, color = red) : # torque corresponding to CMF

Com_new_CMF := complexplot(Fc_new_CMF - I·Tc_new_CMF, logf = 2 ..10, color = green) :

display(F_class_plot, F_ω0_class_plot, F_ωinf_class_plot, T_class_plot, Fc_new_plot, Tc_new_plot, title
    = "new CMF vs. classical CMF") :
display(ComClass, Com_new_CMF, scaling = constrained, labels = [Real_CMF, Imag_CMF]) :
display(Fc_plot, Tc_Nm_plot, Fc_p0_plot, Tc_Nm_ref_plot, title = "force & torque: new vs. classic") :
```

complex plots

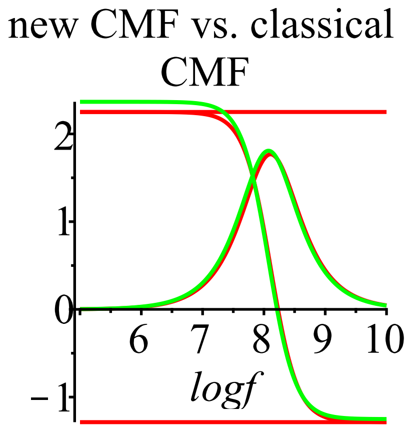

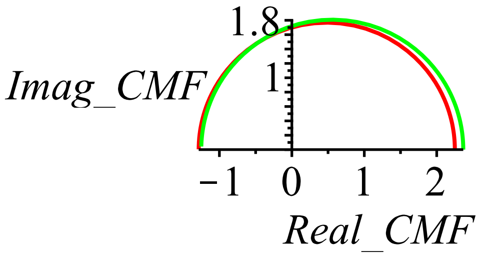

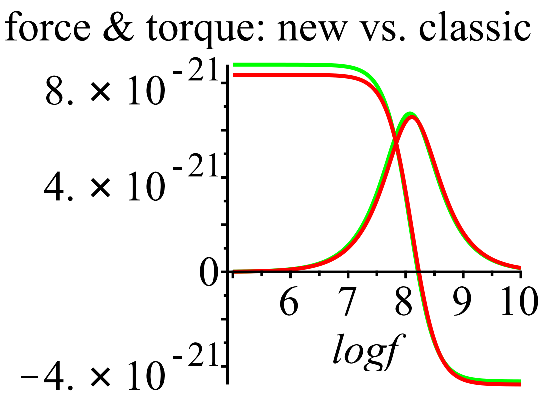

The effective relative permittivity and conductivity in the suspension box (green) are the sum of their active (blue) and reactive (gold) components

Note that the green sum curves reflect the characteristic frequency-dependent decrease in permittivity and the corresponding increase in conductivity of suspensions.

```
ϵS_com_plot := plot(Re(ϵS_com), logf = 5 ..10, color = green) :
ϵS_act_plot := plot(Re(ϵS_act), logf = 5 ..10, color = blue) :
ϵS_react_plot := plot(Re(ϵS_react), logf = 5 ..10, color = gold) :

σS_com_plot := plot(Re(σS_com), logf = 5 ..10, color = green) :
σS_act_plot := plot(Re(σS_act), logf = 5 ..10, color = blue) :
σS_react_plot := plot(Re(σS_react), logf = 5 ..10, color = gold) :

display(ϵS_com_plot, ϵS_act_plot, ϵS_react_plot, title
    = "complex (green), active (blue) and reactive (gold) components of permittivity") :
display(σS_com_plot, σS_act_plot, σS_react_plot, title
    = "complex (green), active (blue) and reactive (gold) components of conductivity") :
```

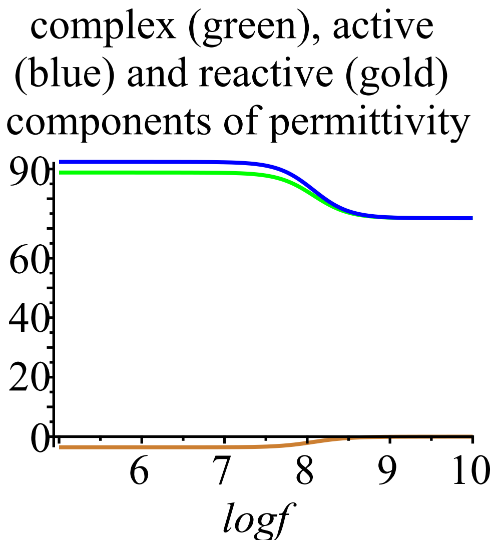

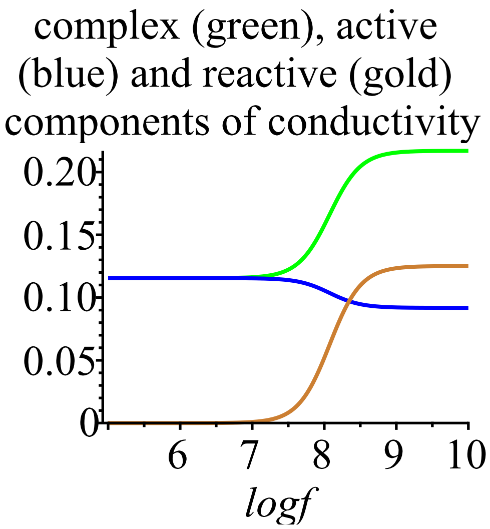

Supplement: Supplementary file 1 [file micromachines-12-00738-s001.zip › micromachines-1245705-supplementary.pdf]
